# Early‐stage diagnosis of nutrient deficiency by chlorophyll fluorescence in maize

**DOI:** 10.1111/plb.70273

**Published:** 2026-07-31

**Authors:** M. Jezek, K.‐H. Mühling, M. Waqas, A. S. Richter, C.‐M. Geilfus, W. Bilger

**Affiliations:** ^1^ Institute of Plant Nutrition and Soil Science Kiel University Kiel Germany; ^2^ State Key Laboratory of Desert and Oasis Ecology, Xinjiang Institute of Ecology and Geography Chinese Academy of Sciences Urumqi China; ^3^ Physiology of Plant Metabolism, Institute for Biosciences University of Rostock Rostock Germany; ^4^ Department of Soil Science and Plant Nutrition Hochschule Geisenheim University Geisenheim Germany; ^5^ Institute of Botany Kiel University Kiel Germany; ^6^ Present address: Lancaster UK

**Keywords:** Anthocyanin index, chlorophyll fluorescence, flavonol index, maize phenotyping, nutrient deficiency

## Abstract

- Rapid, non‐destructive phenotyping is vital for early nutrient deficiency detection in plant research and agriculture. Nitrogen (N) and magnesium (Mg) deficiency are hard to distinguish with instruments measuring only chlorophyll content since both deficiencies lead to loss of chlorophyll.
- This study evaluated the suitability of a commercially available dual‐excitation fluorescence sensor (measuring chlorophyll (Chl) and epidermal UV absorbing compounds) for identifying nutrient deficiencies in maize seedlings.
- Both N and Mg deficiencies in maize seedlings caused a decrease in the Chl index and an increase in the flavonol (Flav) index, although the response of the Flav index was much weaker under Mg deficiency. This happened in spite of a much larger increase in sugar concentrations under Mg deficiency. Furthermore, the nitrogen balance index (NBI) detected N deficiency earlier in leaves developing under nutrient deficiency than in those present before treatment application. Spatiotemporal analysis revealed distinct patterns of Flav index increase: N deficiency caused a marked increase in upper (younger) leaves, whereas Mg deficiency initiated Flav index increases at the tips of lower (older) leaves. Utilizing green‐ and red‐light excitation of chlorophyll to infer epidermal anthocyanins turned out to be not straightforward in nutrient‐deficient maize leaves.
- Taken together, these findings reveal advantages and limitations of Chl fluorescence in diagnosing nutrient stress and underscore the importance of understanding spatial‐temporal nutrient dynamics for accurate early detection. Leaf age should be considered for fertilization decisions based on the NBI. A strategy is suggested how anthocyanins can be detected without problems.

## Graphical abstract

Symptomatic differences between nitrogen and magnesium deficiency in maize seedlings can be detected using chlorophyll fluorescence to measure spatiotemporal changes in leaf chlorophyll and epidermal UV‐absorbing compounds for early diagnosis and differentiation of nutrients.

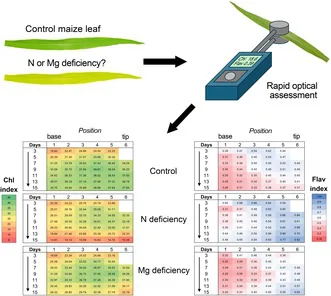

## INTRODUCTION

Optimal fertilizer management practice involves applying the right nutrient source at the right concentration combined with the right placement to the root (*i.e*., contact fertilization) to guarantee optimal crop growth, soil health and decreased pollution of the environment (Senbayram *et al*. 2015). This strategy requires suitable devices for monitoring the nutritional status of crops throughout the growing period to assist the demand‐oriented application of fertilizers. Several non‐destructive and rapid methods have been developed to assess the crop nitrogen (N) status. N is the primary plant nutrient with a significant impact on yield formation and usually has the highest application rate in agriculture. N is needed for photosynthesis and protein biosynthesis, and N shortage consequently impairs crop yield and quality by affecting these essential processes (Waqas *et al*. 2023a). The first visual N deficiency symptom is leaf chlorosis caused by decreasing chlorophyll (Chl) content, and a robust linear relationship between leaf N and Chl content was found for many crops (Evans 1989). Furthermore, a low N status increases the biosynthesis of carbon‐based and N‐free secondary compounds, including flavonoids (Dixon & Paiva 1995; Lea *et al*. 2007; Negwer & Bilger 2026). Flavonoids such as flavonols and anthocyanins are mainly synthesized in epidermal cells in response to abiotic stress (Geilfus 2019). Part of their ecophysiological function, for example the attraction of pollinators, is understood whereas their physiological significance during nutrient deficiency is not consensually resolved. Owing to their light‐absorbing properties, flavonoids are supposed to protect plants against the detrimental effects of UV radiation (Caldwell *et al*. 1983; Stapleton & Walbot 1994). Furthermore, specific flavonoids have antioxidant potential and might scavenge stress‐induced reactive oxygen species (Pollastri & Tattini 2011; Waqas *et al*. 2023b; Agati *et al*. 2025).

The relationship between N availability, flavonoid synthesis and crop growth has also been explained in the ‘carbon‐nutrient balance hypothesis’ (Bryant *et al*. 1983) and the ‘growth‐differentiation balance hypothesis’ (Herms & Mattson 1992). These theories regard flavonoids synthesized under N deficiency as carbon storage compounds when N limitation affects biosynthetic processes and restricts growth combined with ongoing photosynthetic CO_2_ assimilation. Further proposed functions are the defence against herbivores or osmoregulation (Chalker‐Scott 2002; Gould 2004). Several transcription factors and enzymes of the flavonoid biosynthesis pathway are induced by the external application of sugar (Teng *et al*. 2005; Solfanelli *et al*. 2006). Because sugar accumulation is known to occur under some nutritional disorders (Cakmak *et al*. 1994a; Hermans *et al*. 2006; Pourtau *et al*. 2006; Verbruggen & Hermans 2013), sucrose or other hexoses might act as signalling elements in nutrient deficiency‐induced flavonoid production. Indeed, increased cellular sugar content stimulated flavonoid biosynthesis during high‐light acclimation (Zirngibl *et al*. 2023).

Both decreasing Chl and increasing flavonoid contents are potential indicators for N deficiency (Cartelat *et al*. 2005; Cerovic *et al*. 2015). Non‐destructive hand‐held leaf‐clip devices have been designed to estimate Chl and flavonoid content in leaves. These instruments can be used for rapid phenotyping, screening, and early diagnosis of nutrient shortages in the field. The ‘Dualex Scientific+™’ simultaneously assesses the Chl content in leaves and the epidermal contents of UV‐A light absorbing compounds, consisting largely, but not exclusively, of flavonols (Flav) as well as those of anthocyanins (Anth) on the same leaf spot by measuring Chl fluorescence excited with UV‐A, green and red light. In this device, the Chl fluorescence excited with red light, which is not absorbed by flavonoids, serves as a reference and is compared to Chl fluorescence induced at wavelengths that are absorbed by epidermal metabolites, such as flavonoids, before reaching the Chl in the mesophyll. The primary flavonoids of interest are flavonols and anthocyanins, each with distinct spectral properties. Flavonols have an absorption maximum in the UV‐A, whereas that of anthocyanins lies in the green spectral region. By utilizing these absorption characteristics, the device can distinguish between the contents of these metabolites in the leaf epidermis.

In theory, different contents of epidermal flavonols or anthocyanins influence Chl fluorescence excited with UV‐A (Flav) or green light (Anth), respectively (Agati *et al*. 2005). Probably with a lesser efficiency, also anthocyanins (Nichelmann & Bilger 2017), and presumably UV‐A absorbing compounds as well, located in the mesophyll reduce chlorophyll excitation. In contrast, the reference excitation light (red, ~650 nm) can freely penetrate the epidermis; hence, Chl fluorescence excited at this wavelength is independent of the amount of epidermal secondary compounds (Bilger *et al*. 1997). Next to providing indices for epidermal light‐absorbing compounds, the Dualex instrument calculates a so‐called nitrogen balance index (NBI), which is the ratio of the Chl and Flav indices and is assumed to be related to metabolic nitrogen/carbon allocation (Cerovic *et al*. 2015). Nitrogen nutrition has opposite effects on foliar flavonol and chlorophyll contents, accentuating differences in the NBI between N‐sufficient and N‐deficient plants better than the two indices alone (Cartelat *et al*. 2005). Therefore, the NBI is conceived as a more precise method to get earlier information about the N status of crops and could enable real‐time site‐specific N fertilization (Cartelat *et al*. 2005; Force‐A 2015). However, declining leaf Chl content and the flavonoid biosynthesis pathway induction are not N‐specific deficiency responses, but can also occur under magnesium (Mg) deficiency (Kobayashi *et al*. 2013; Jezek *et al*. 2015). This overlap in responses could lead to the misinterpretation of deficiency symptoms, complicating the use of Dualex measurements for making precise N‐fertilization decisions at the early stages of crop growth.

In this study, the suitability of the Dualex device for early‐stage discrimination of nutrient deficiency was tested in hydroponically grown maize seedlings. The results are particularly relevant for fertilization management and agricultural research analysing the plant response to different types of nutrient stress. Specifically, observing how plants react to nitrogen (N) and magnesium (Mg) deficiencies, which both lead to chlorophyll loss, can provide insights into whether flavonoid biosynthesis is a general stress response or specific to certain nutrient deficiencies.

The objectives of this work were to (i) evaluate the discriminative power of the Dualex device for the early‐stage distinction between nutrient deficiencies with similar visual symptoms in maize seedlings, (ii) visualize the ontogenetic progression of UV‐A absorbing compound and anthocyanin accumulation in N‐ or Mg‐deficient maize seedling leaves, respectively, and (iii) analyse the connection between flavonoid biosynthesis and soluble sugar contents in nutrient‐deficient maize seedlings.

## MATERIAL AND METHODS

### Plant growth conditions

Maize (*Zea mays* L. cv. Susann, Nordsaat Saatzucht, Langenstein, Germany) plants were grown hydroponically in a climate chamber (16/8 h day/night; 25 °C/18 °C; 60% relative humidity; 200 μmol m^−2^ s^−1^ PAR). Seeds were soaked in 2 mM aerated CaSO_4_ solution for 1 day and germinated in plastic foam sandwiches moistened with 2 mM CaSO_4_. Five‐day‐old seedlings were transferred into 5 l plastic vessels (four plants per vessel with four vessels per treatment) with 25% strength nutrient solution with different N or Mg concentrations (Table 1), respectively, which was raised stepwise to 100% strength within 3 days. The day of transfer into the plastic vessels is henceforth called ‘day of planting’ and the timeline is given as ‘days after planting’ (DAP). The nutrient solution was exchanged weekly in order to avoid nutrient depletion. Three experimental groups with different nutrient supply were cultivated in four independent biological replicates.

**Table 1:** Composition of the nutrient solution of hydroponically grown maize grown under optimal (Control), nitrogen‐deficient (N def.; 4% N of Control) or magnesium‐deficient (Mg def.; 4% Mg of Control) conditions.

nutrient salt	control	Mg def.	N def.
	mmol l^−1^	mmol l^−1^	mmol l^−1^
Ca(NO_3_)_2_	1.3	1.3	0
NH_4_NO_3_	0.7	0.7	0.08
CaCl_2_	2	2	3.3
K_2_SO_4_	1	1	1
KH_2_PO_4_	0.2	0.2	0.2
MgSO_4_	0.5	0.02	0.5
	μmol l^−1^	μmol l^−1^	μmol l^−1^
Fe‐EDTA	200	200	200
H_3_BO_3_	5	5	5
MnSO_4_	2	2	2
ZnSO_4_	0.5	0.5	0.5
CuSO_4_	0.3	0.3	0.3
(NH_4_)_2_Mo_7_O_24_	0.01	0.01	0.01

### Optical measurements of leaf pigments

Chlorophyll (Chl), flavonol (Flav), anthocyanin (Anth) and nitrogen balance index (NBI) measurements were conducted using the polyphenol and chlorophyll meter ‘Dualex Scientific+™’ (FORCE‐A, Orsay, France). Readings were taken every other day, starting on the third emerging foliage leaf of 8‐days‐old seedlings three DAP. Recordings on the fourth and fifth foliage leaves started five DAP and 11 DAP, respectively, according to their appearance (Figs 5D and 6D). Four vessels with four plants each per treatment were analysed, resulting in 16 leaves per leaf level per experimental group. Five to six measurements – depending on the leaf age and size – were made along the leaf lamina, avoiding the midribs, if allowed by the leaf size (Figs 5D and 6D). For very few initial time points and leaf levels, only two or three plants per pot were analysed due to the small leaf size of some plants. Readings were made on the adaxial leaf side. For analysis of temporal courses, measurements along the leaf blade were averaged.

The nitrogen balance index (NBI) is defined as the ratio between the Chl index and the Flav index.

$$\mathrm{NBI}=\frac{\mathrm{Chl}\;\text{index}}{\text{Flav index}}$$

### Harvest

Analysed leaves of 18‐days‐old seedlings were harvested separately 15 days after planting. For further analysis, leaf material was dried at 60 °C for 2 days and finely ground to a powder using a ball mill.

### Analysis of soluble sugars

Soluble sugars were extracted by hot water extraction. Five milligram of finely ground leaf material was dissolved in double distilled (dd) H_2_O and boiled at 100 °C for 5 min in a water‐bath with proper mixing in between. Samples were cooled on ice for 30 min, centrifuged (4 °C, 12,000 rpm, 10 min), and the supernatant was collected and diluted 1:5 with ddH_2_O, if necessary. Samples were cleaned‐up with chloroform and run over C18 columns (Strata spe. 8B‐S001‐DAK, Phenomex, Torrance, CA, USA). Sucrose, glucose and fructose concentrations were measured by anion exchange chromatography using an ICS‐5000 system equipped with a Carbo Pac PA‐100 column and an integrated amperometric detector (Dionex, Sunnyvale, CA, USA).

### Statistics

Data of Dualex measurements were statistically analysed using R statistical package software version 3.0.2 (The R Foundation for Statistical Computing, Vienna, Austria). Effects of treatments were tested by using one‐way ANOVA with a Tukey‐HSD test as *post hoc* test. Spatiotemporal analysis of the data was visualized using Microsoft Excel 2013 (Microsoft Corporation, Redmond, Washington, USA).

## RESULTS

### Dualex measurements

**Fig. 1:**
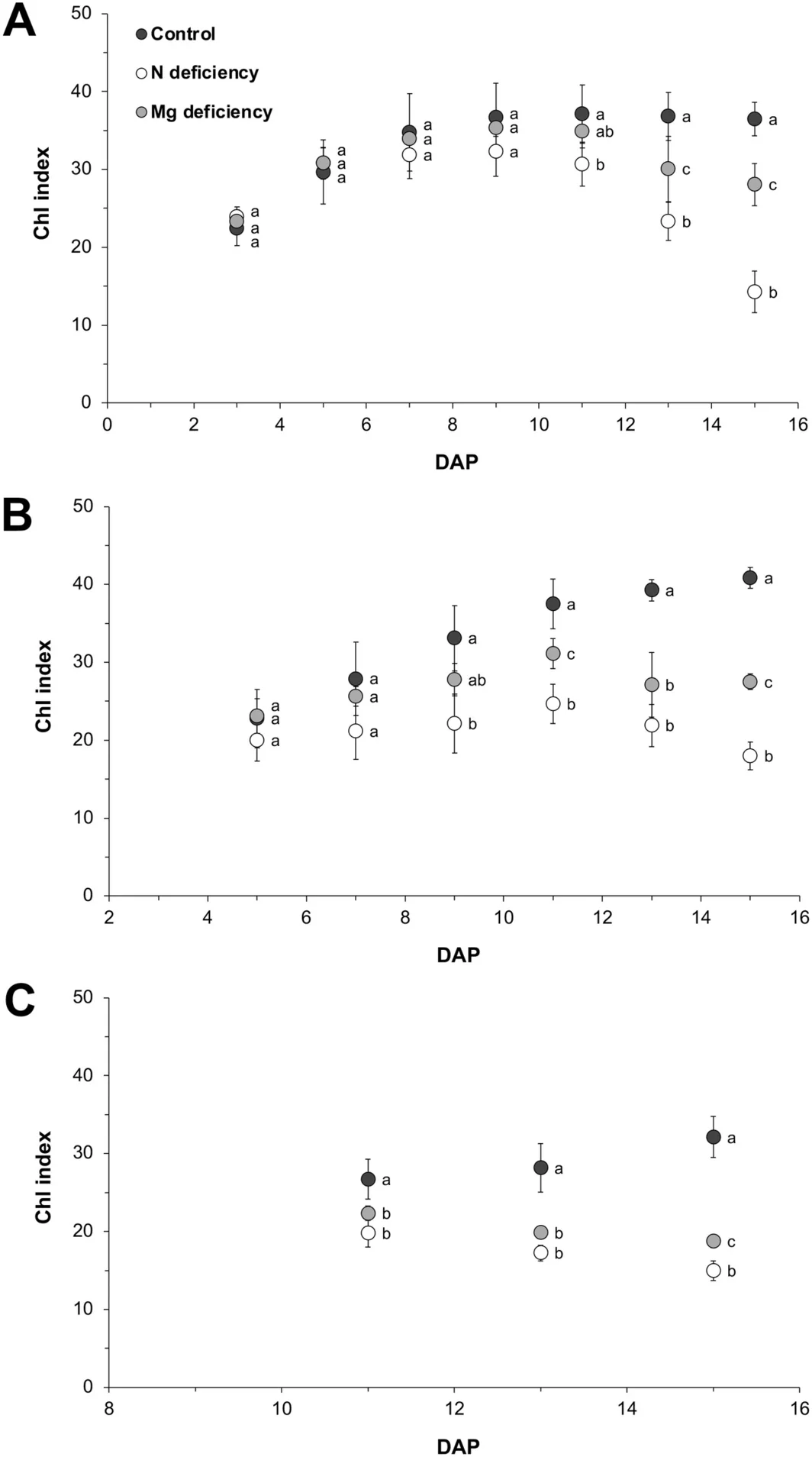
Time course of the Chl index of (A) leaf 3, (B) leaf 4 and (C) leaf 5. Control plants were grown under optimal nutrition; N and Mg deficiency was induced by reducing the N or Mg concentration, respectively, in the nutrition solution to 4% of the control. Measurements were taken on four plants per vessel. Five to six measurements were taken along the leaf blade and averaged. Means ± SD; n = 4. Small letters indicate significant differences between treatments at a measuring date (one‐way ANOVA with Tukey‐HSD test, *P* ≤ 0.05). DAP, days after planting.

Five to six measurements, depending on the leaf age, were taken along the leaf blade and averaged. A Chl index increasing with leaf age was measured in all leaves of control plants. In contrast, the Chl index increased in leaf 3 of N‐ and Mg‐deficient plants during the first half of the experiment but stagnated and decreased in the following (Fig. 1). For N and Mg deficiency, the Chl index values were different compared to the control at day 11 and day 13 after planting (DAP), respectively. The Chl index in leaf 5 of nutrient‐deficient plants was overall and in leaf 4 from DAP 11 on significantly lower than the control and declined until the end of the experiment. The chlorophyll decrease was more pronounced in N‐deficient plants than in Mg‐deprived ones (Fig. 1).

**Fig. 2:**
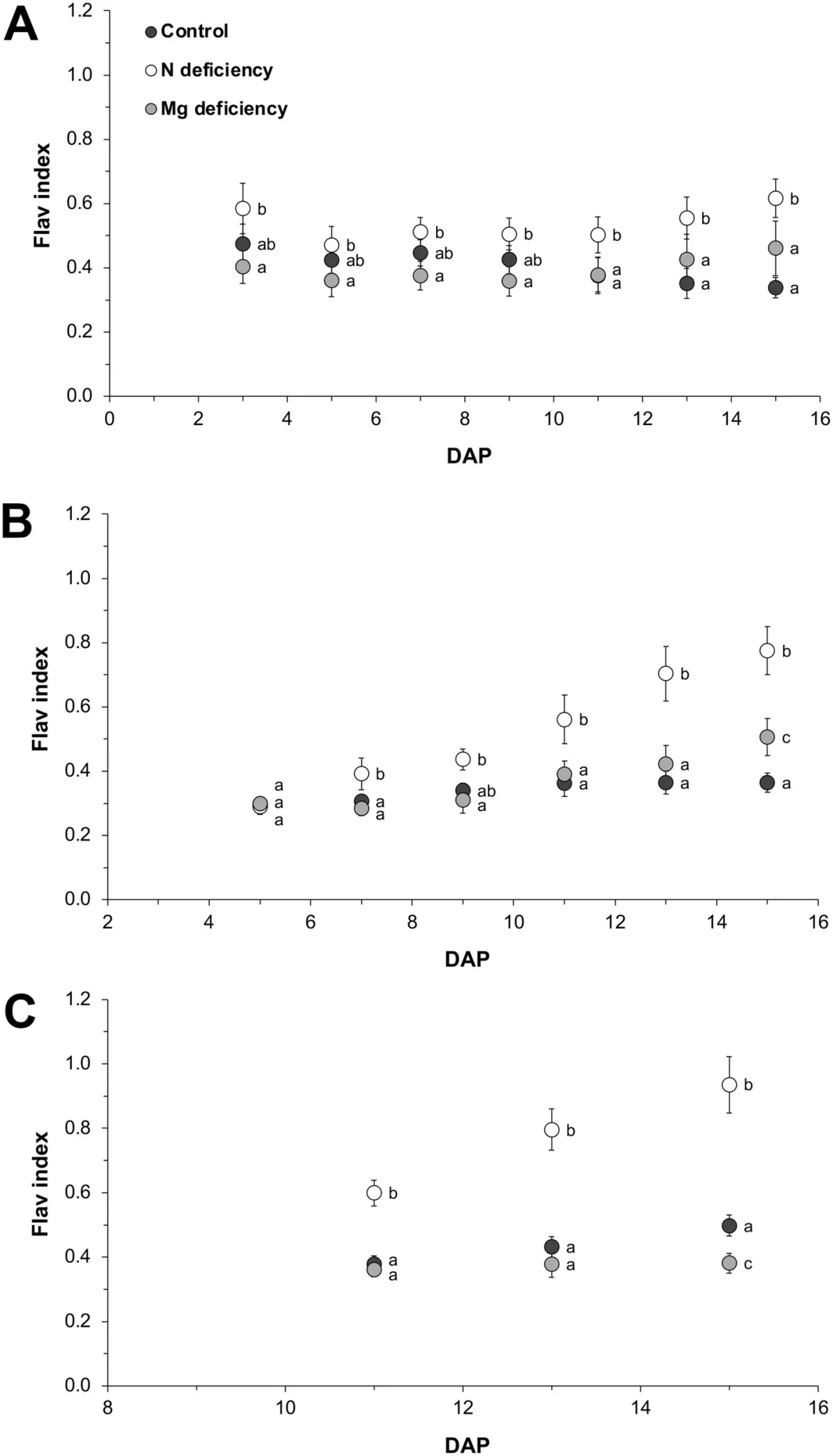
Time course of the Flav index of (A) leaf 3, (B) leaf 4 and (C) leaf 5. Control plants were grown under optimal nutrition; N and Mg deficiency was induced by reducing the N or Mg concentration, respectively, in the nutrition solution to 4% of the control. Measurements were taken on four plants per vessel. Five to six measurements were taken along the leaf blade and averaged. Mean ± SD; n = 4. Small letters indicate significant differences between treatments at a measuring date (one‐way ANOVA with Tukey‐HSD test, *P* ≤ 0.05). DAP, days after planting.

Only minor changes were found in the Flav index averaged over the whole leaf blade in control plants in the course of the experiment (Fig. 2). In contrast, the Flav index increased markedly over time under N shortage. Flav values of all leaves were twice as high as in control leaves at harvest (Fig. 2). Low Mg supply increased the Flav index only in leaf 4 on day 15 (Fig. 2).

**Fig. 3:**
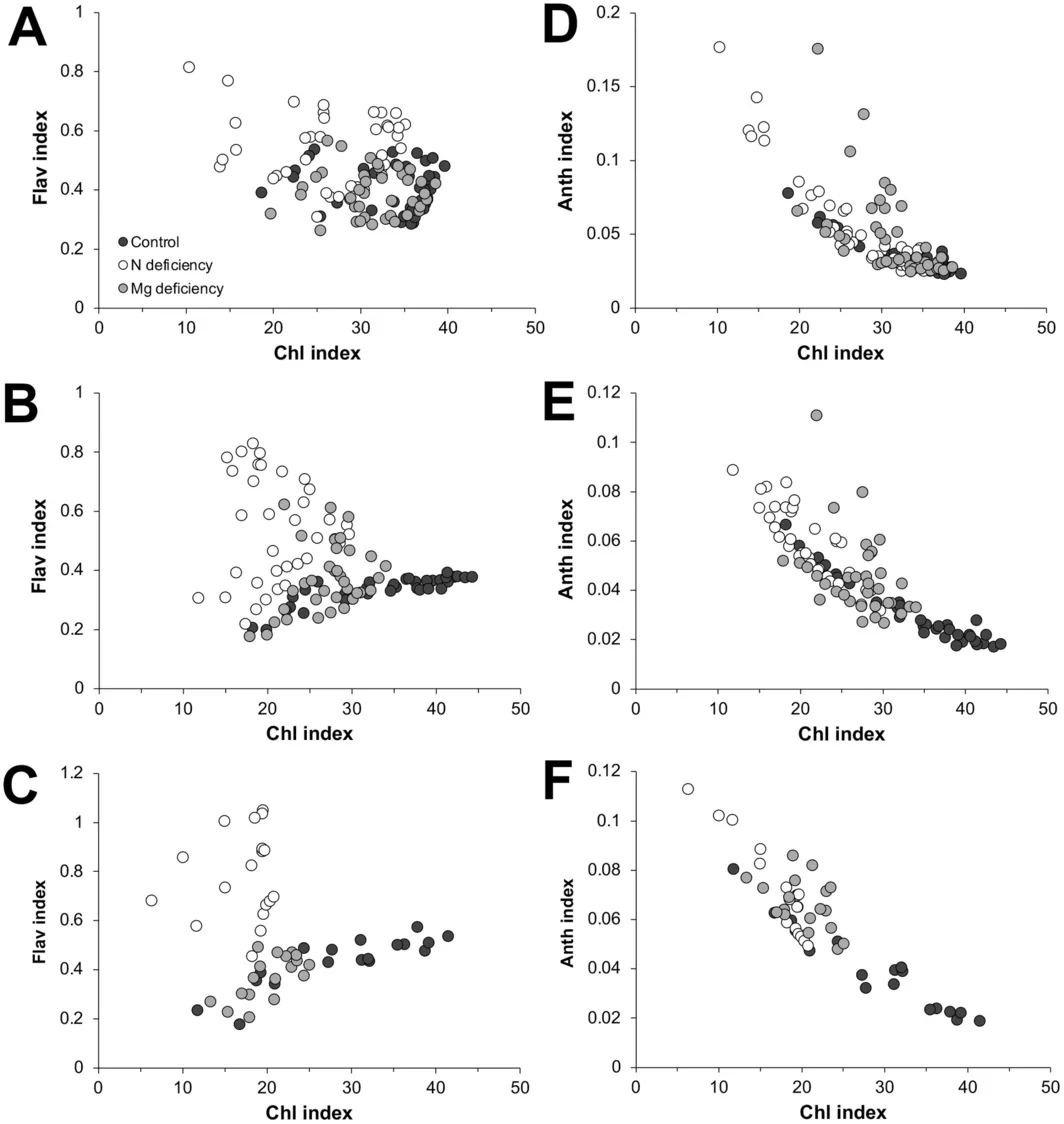
Flav index in (A) leaf 3, (B) leaf 4 and (C) leaf 5 and Anth index in (D) leaf 3, (E) leaf 4 and (F) leaf 5 of maize seedlings as a function of the simultaneously determined Chl index. Dualex measurements were taken along the leaf blade and mean values of four plants at individual measuring locations were correlated (see Figs 1 and 2 for time course of Chl and Flav indices).

While the response of the maize leaves to the two different nutrient deficiencies was qualitatively similar with decreases in chlorophyll content and increases in UV‐A absorbing compounds, the relationships between both effects were different for the two treatments (Fig. 3). In leaves of all ages, data points from N deficiency were clearly shifted to lower chlorophyll values with higher Flav values (Fig. 3A–C). This trend was stronger the younger the leaves. On the other hand, data from Mg deficiency coincided in most cases with those of the control leaves. The Anth index showed a much smaller response to the treatments, varying between 0.02 and 0.1 with few exceptions (Fig. 3D–F). There was a general trend for an increasing Anth index with decreasing chlorophyll index, irrespective of the treatment. This relationship probably results from optical changes affecting the ratio between light absorption by chlorophyll at the green and red wavelength regions. An exception is data points under Mg deficiency especially in leaf 3 at chlorophyll index values below 30, where Anth values increased above the general relationship. These data points might indicate specific anthocyanin formation during beginning senescence towards the end of the experiment, and purple colouration of these leaves was clearly visible (Fig. S1B). Hence, nutrient deficiencies appear to influence chlorophyll and flavonoid levels, with nutrient‐specific impacts on pigment composition (Fig. 3).

**Fig. 4:**
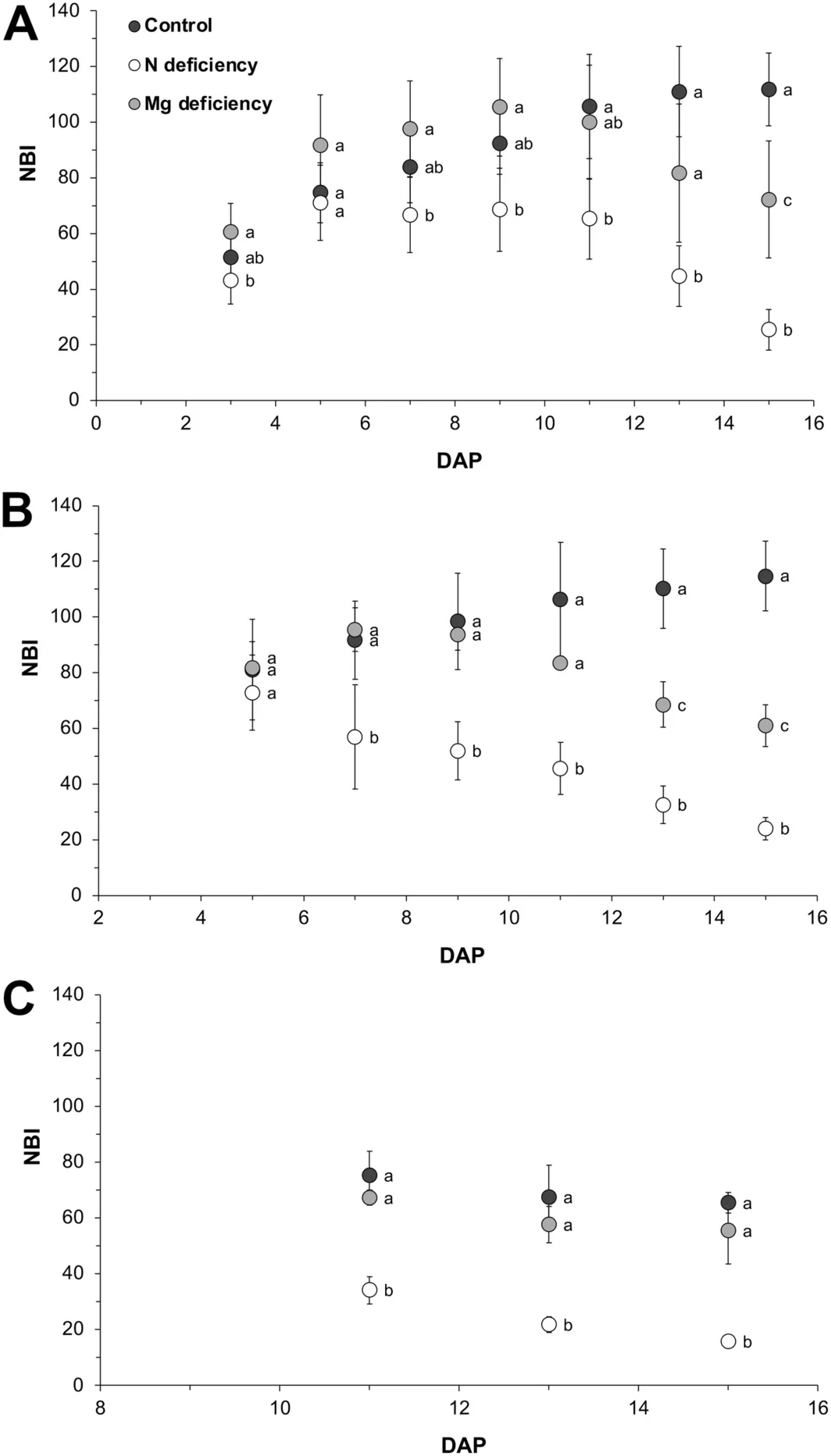
Time course of the NBI index of (A) leaf 3, (B) leaf 4 and (C) leaf 5. Control plants were grown under optimal nutrition; N and Mg deficiency was induced by reducing the N or Mg concentration, respectively, in the nutrition solution to 4% of the control. Measurements were taken on four plants per vessel. Five to six measurements were taken along the leaf blade and averaged. Mean ± SD; n = 4. Small letters indicate significant differences between treatments at a measuring date (one‐way ANOVA with Tukey‐HSD test, *P* ≤ 0.05). DAP, days after planting.

The nitrogen balance index NBI is defined as the ratio of the Chl and Flav index and has been proposed as an indicator for nitrogen deficiency (Cartelat *et al*. 2005). Figure 4 shows that from day 7 on, NBI of N‐deficient leaves showed significantly lower values than the control in all leaves, but this effect was more pronounced in the two younger leaves. In contrast to the N deficiency treatment, NBI values under Mg deficiency differed much less from the control and if so, only towards the end of the treatment period (Fig. 4).

**Fig. 5:**
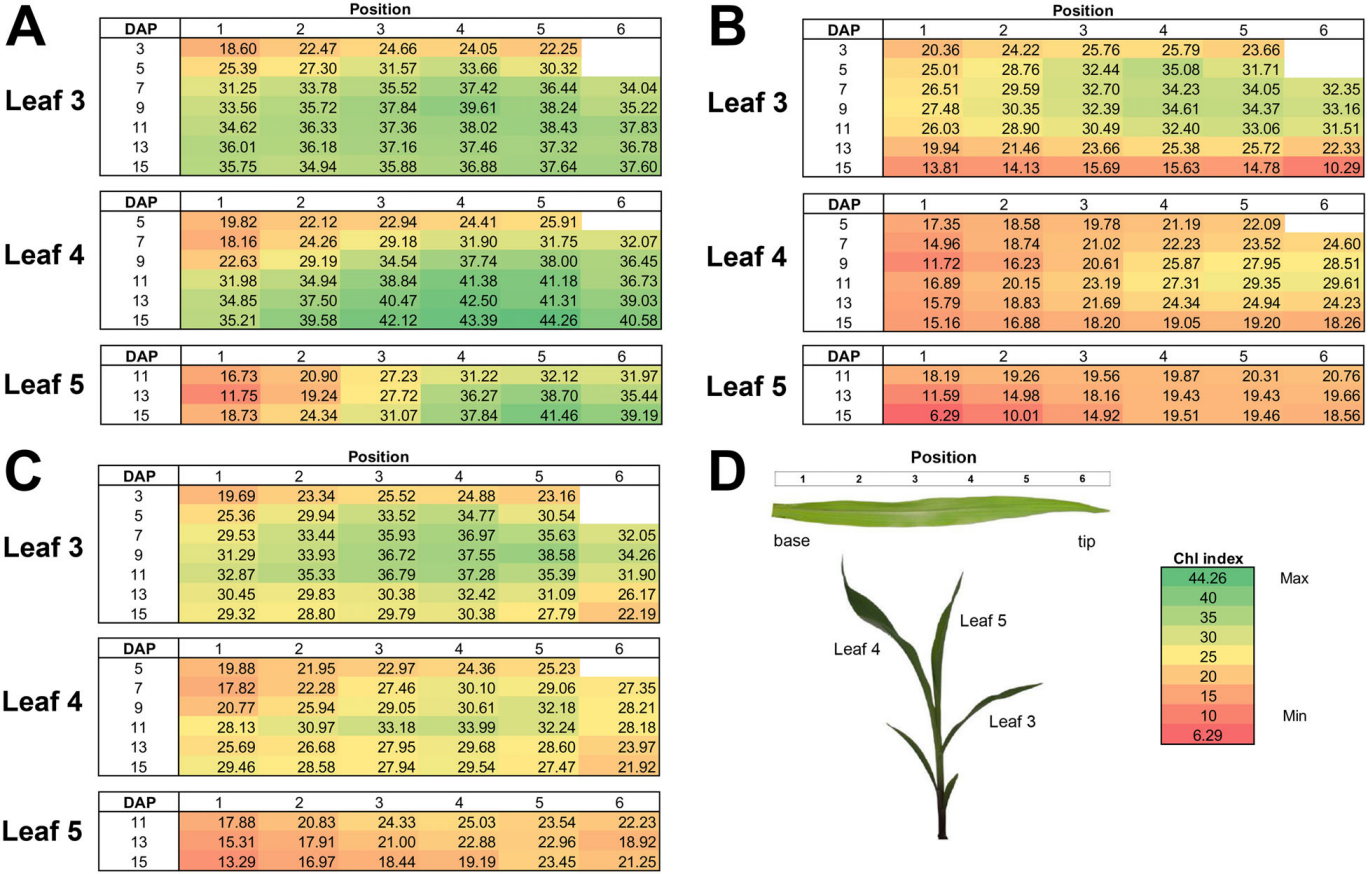
Spatiotemporal courses of Chl index of leaves 3–5 from (A) control, (B) N‐deficient and (C) Mg‐deficient maize seedlings. The colour gradient from green (maximum) to yellow (intermediate) to red (minimum) shows the varying range of the Chl index. Control plants were grown under optimal nutrition; N and Mg deficiency was induced by reducing the N or Mg concentration, respectively, in the nutrition solution to 4% of the control (n = 4). Measurements were taken on four plants per vessel. (D) Illustration of the measuring procedure. Dualex measurements were made at five to six leaf spots along the leaf blade – depending on the leaf age and size – starting from the leaf base. Leaves number 3–5 of maize seedlings were analysed.

**Fig. 6:**
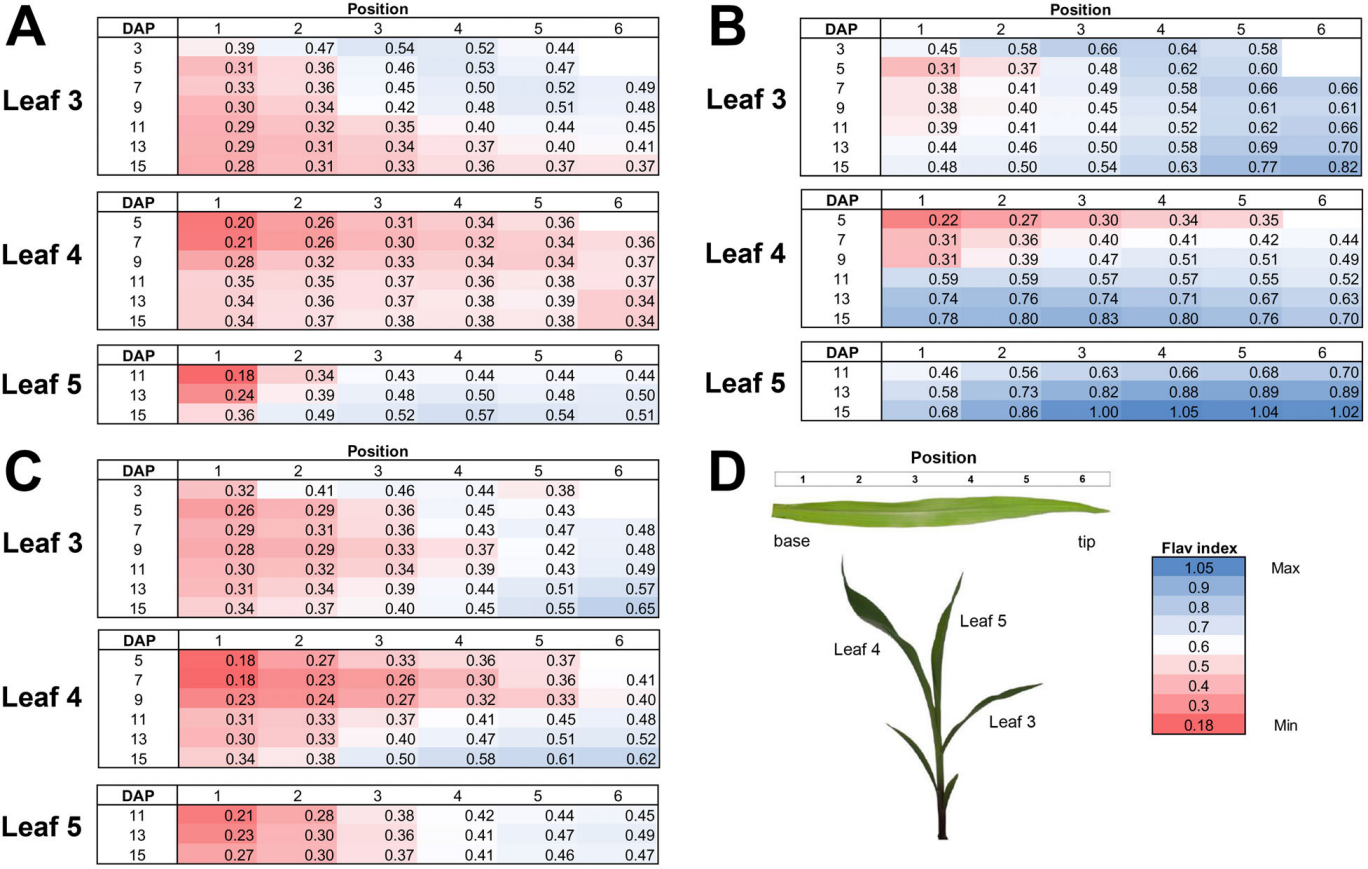
Spatiotemporal courses of Flav index of leaf 3–5 from (A) control, (B) N‐deficient and (C) Mg‐deficient maize seedlings. The colour gradient from blue (maximum) to white (intermediate) to red (minimum) shows the varying range of the Flav index. Control plants were grown under optimal nutrition; N and Mg deficiency was induced by reducing the N or Mg concentration, respectively, in the nutrition solution to 4% of the control. Measurements were taken on four plants per vessel. (D) Illustration of the measuring procedures. Dualex measurements were made at five to six leaf spots along the leaf blade – depending on the leaf age and size – starting from the leaf base. Leaves number 3–5 of maize seedlings were analysed.

Grass leaves are growing from the base, causing a pronounced cellular age gradient along the leaf lamina. The spatiotemporal visualization of the measurements provided information about changes in the Chl and Flav indices along the leaf lamina over time (Figs 5 and 6). Irrespective of the nutritional status, longitudinal gradients of both Chl and Flav indices were measured along the leaf blade, increasing from the base to the tip. The Chl index was generally slightly lower in the leaf tip. Furthermore, it was shown that the Flav index of Mg‐deficient maize leaves increased predominantly at the tip in leaves 3 and 4 (Fig. 6C). This was also true for leaves 3 and 5 of plants with low N supply where the Flav index was higher at the tip than at the base of the leaf; however, the Flav index in leaf 4 of N‐deficient plants was high at the leaf base as well on the last 3 days of measurements (Fig. 6B).

### Sugar measurements

**Fig. 7:**
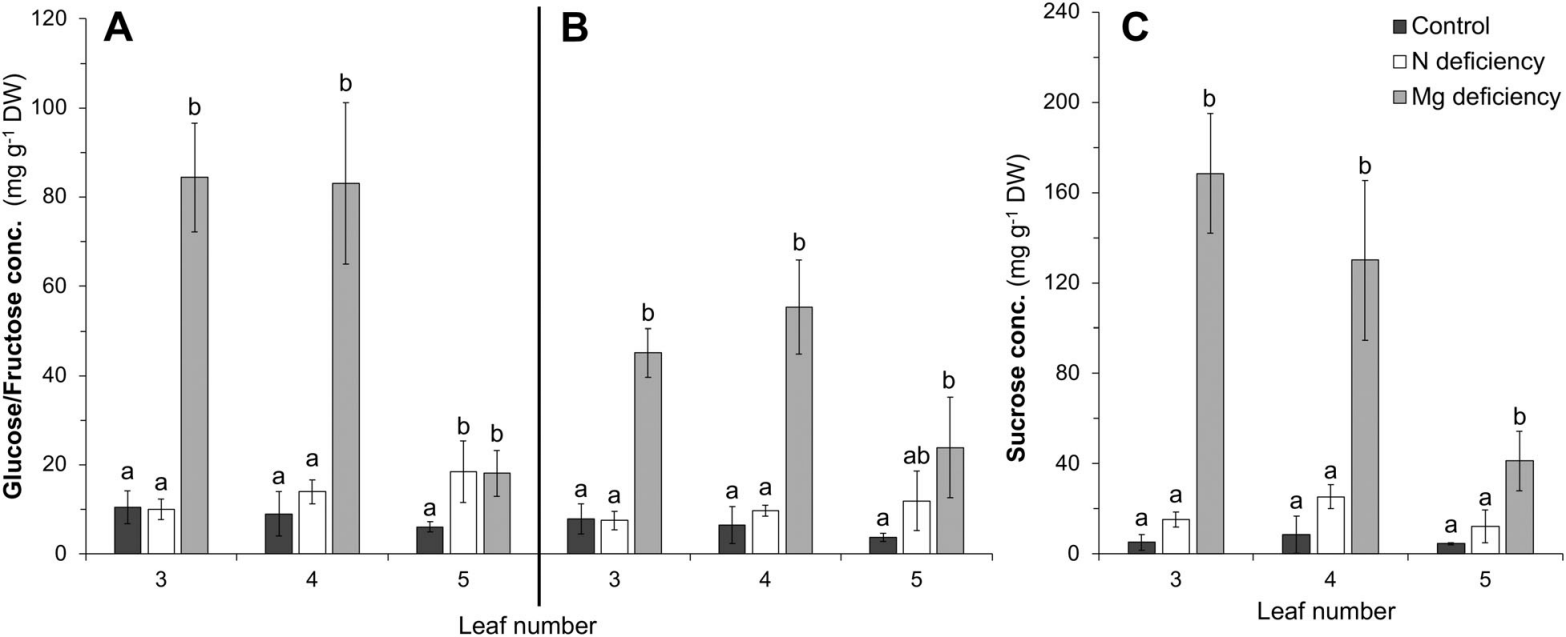
Concentrations of soluble glucose (A), fructose (B) and sucrose (C) in maize leaves. Control plants were grown under optimal nutrition; N and Mg deficiency was induced by reducing the N or Mg concentration, respectively, in the nutrition solution to 4% of the control. Mean ± SD; n = 4. Small letters indicate significant differences between treatments at a measuring date (one‐way ANOVA with Tukey‐HSD test, *P* ≤ 0.05).

The content of soluble sugars was measured in extracts from whole leaves after the harvest of plants at 15 DAP. Deficient Mg supply resulted in markedly increased contents of soluble glucose, fructose and sucrose in all leaves (Fig. 7). Under Mg deficiency, sucrose levels were up to 32 times as high as in the control in leaf 3, reaching almost 170 mg g^−1^ DW (Fig. 7C). Nitrogen‐deficient maize seedlings accumulated significantly more glucose in leaf 5 and sucrose in leaves 3 and 4 than the control (Fig. 7A,C). However, sugar concentration rises were far less pronounced than under Mg deficiency.

## DISCUSSION

### Evaluation of Dualex measurements for early‐stage detection of nutrient deficiency

The present study aimed to evaluate the suitability of the ‘Dualex Scientific+™’ for early‐stage discrimination between N and Mg deficiency in maize seedlings. Evaluating non‐destructive rapid phenotyping techniques is of agronomic interest because they can assist fertilizer management to take the correct measures against nutrient deficiency.

The suitability of the Dualex device for measuring leaf contents of chlorophyll and UV‐absorbing compounds has been intensively tested, and good correlations between the Flav index and extracted UV‐absorbing phenolic compounds were found in diverse crops and under different N levels (Goulas *et al*. 2004; Cartelat *et al*. 2005; Pfündel *et al*. 2007; Cerovic *et al*. 2012). Our data showed a general significant response of the Chl, Flav and NB indices to the induced nutrient deficiency, supporting the general usefulness of the device.

The response to nutrient deficiency developed over time, with significance of the differences of the Chl and Flav indices among the treatments starting at DAP 7. As expected, chlorophyll content in N‐ and Mg‐deficient leaves was lower than in control plants and declined with leaf ageing (Figs 1 and 5B,C). Since the photosynthetic machinery accounts for over 50% of leaf N content (Evans 1983, 1989), Chl is degraded during N deficiency, and N is relocated to growing tissue. Similarly, the photosynthetic apparatus, including Chl, is dismantled under Mg deficiency due to negative feedback from accumulating photoassimilates (Hermans & Verbruggen 2005).

Transiently higher Flav values were found in control leaf 3 (Fig. 6A). A juvenile flavonoid accumulation is known from anthocyanins (Murray & Hackett 1991; Chalker‐Scott 1999) and might display a photomorphogenic ‘high irradiance response’ presumably for photoprotection of the developing photosynthetic apparatus (Chalker‐Scott 1999). The results of our study indicate that UV‐A absorbing compounds are also temporarily increased at the onset of leaf emergence in maize seedlings independent of the nutritional status. Decreasing Flav index over time has also been measured in flag leaves of field‐grown wheat under optimal N conditions before the onset of senescence, indicating turnover of epidermal UV‐A absorbing compounds (Cartelat *et al*. 2005) or, alternatively, dilution of a constant amount of these compounds by cellular expansion growth.

The Flav index of all analysed leaves in N‐deprived maize increased strongly over time (Figs 2 and 6B). Transcriptional up‐regulation of critical enzymes of the flavonoid biosynthesis pathway in response to N depletion has been thoroughly documented in *Arabidopsis thaliana* (Lillo *et al*. 2008). Tomato plants grown under limited N conditions show a marked increase in foliar flavonols but not in fruits (Stewart *et al*. 2001; Bénard *et al*. 2011). UV‐A absorbing compound accumulation was most pronounced in the uppermost leaf of maize seedlings under N deficiency, which may have been caused by a more severe deficiency in the newly developing leaves, as indicated by their Chl indices, which never reached the highest levels observed under nutrient deficiency in the third leaf. In addition, increasing irradiance with plant height may have further contributed to enhanced formation of UV‐A absorbing compounds in the younger leaves.

Increases in the Flav values were found in maize leaves 3 and 4 also in response to Mg shortage, albeit to a smaller extent than those observed under N deficiency (Fig. 2). Magnesium deficiency is known to induce foliar anthocyanin accumulation (Kobayashi *et al*. 2013). However, up‐regulation of the flavonol biosynthesis pathway has until now not been shown. Cartelat *et al*. (2005) found a high correlation of Chl and Flav indices with N content in wheat leaves and considered both values as probes for crop N nutrition. However, the results of our study reveal that also Mg deficiency leads to a simultaneous decline in Chl values and at least a small increase in the Flav index. For a proper utilization of an optical system to detect a specific nutrient deficiency, it is desirable that the symptoms are nutrient specific. When the Flav and Chl indices were plotted against each other, the data points from the two deficiencies were separated from each other, those from N deficiency lying at higher Flav and lower Chl values. This observation would allow a distinction between both deficiencies, but further experiments, for example with different nutrient dosages and therefore intensities of nutrient stress, are needed to corroborate this finding. Further differences between N and Mg deficiency became obvious when the location of the measuring points along the leaf lamina was included in the analysis. As a phloem‐mobile nutrient, Mg is assumed to be translocated to growing tissue starting from the oldest part of source leaves (Senbayram *et al*. 2015). Accordingly, with increasing duration of the Mg deficiency, the Chl index showed a stronger decrease at the tip of leaves 4 and 5, whereas the Flav index displayed the opposite behaviour (Fig. 6). This base to tip gradient was less pronounced in the control and under N deficiency, especially when leaf 4 was considered. The generality of these patterns should be confirmed in further analyses.

Wagner *et al*. (2003) supposed that leaf gradients of epidermal UV‐absorbing compounds might reflect different sun exposure of distinct leaf regions in barley under optimal nutrition. Hence, UV‐screening compounds accumulated preferentially in the middle part of planophile barley leaves. Our observation of the highest Flav index in the vertically oriented youngest leaf (Figs 2C and 6B) may also partially be explained by a higher light exposure. Under field conditions, differential solar irradiation of the leaves may, in addition, influence the gradient of the Flav index along the leaf lamina.

### Practical usage

What conclusions can be drawn for the practical usage of the Dualex instrument for the detection of nutrient deficiency and the distinction between N and Mg deficiency? First, as initiated by Cartelat *et al*. (2005), the NBI is the most sensitive indicator, integrating the response of chlorophyll and UV‐A absorbing compounds. It reacted stronger to N deficiency than to Mg deficiency. Second, the distinction between both deficiencies became obvious when Flav values were plotted as a function of the determined chlorophyll content (Fig. 3). The relationship between Flav and Chl indices was clearly steeper under N deficiency due to a comparatively stronger response of the Flav index, whereas the data points from Mg deficient leaves collocated largely with those from the control. Third, leaf development should be considered as this tendency was dependent on leaf age, that is, duration of the exposure to nutrient deficiency, and location along the leaf lamina. During growth, the chlorophyll index showed a strong increase in the first days, leading to an increase in NBI until DAP 7. Leaves developing in the presence of nutrient deficiency showed more severe symptoms, which further increased in strength in all leaves with the duration of the deficiency and the Dualex device is obviously able to quantify this tendency. Hence, the practical user should not rely on measurements at a single time point, but consider the development of the data over several days. Fourth, for the interpretation of the measuring results, it is also important to consider the measuring location on the leaf. There are existing gradients along the leaves for all three indices and closer to the tip of the leaves, the reaction to nutrient deficiency tended also to be stronger. Therefore, for the practical application, it is important to take into account the measuring location along the leaves and the relative leaf age. For the youngest leaves close to their tip, the strongest responses are to be expected. When these precautions are taken, it seems possible to detect with a delay of a few days the development of an N or Mg deficiency and with further careful measurements and evaluation, a distinction between both appears possible.

The described experiments were done under laboratory conditions. In the field, higher Flav values are to be expected due to the exposure of the leaves to high sunlight including also UV‐B radiation. There exist several tests of the NBI under field conditions, which showed that it still indicates nitrogen deficiency (Cartelat *et al*. 2005; Tremblay *et al*. 2007; Cerovic *et al*. 2015). The interplay with other mineral deficiencies under field conditions remains to be investigated. For the practitioner, also the response of the indices to nitrogen fertilization after the detection of deficiency may be important. We did not address this problem in our experiments, but would suggest restricting the following Dualex measurements to the youngest developing leaves, since in our hands, UV‐A absorbing compounds have shown a low turnover rate.

### Anthocyanin measurement

The Dualex model, ‘Dualex Scientific+™’, allows epidermal anthocyanin content to be measured by calculating an Anth index from Chl fluorescence measurements upon green and red light excitation. Since anthocyanins are synthesized in response to several nutrient deficiencies (reviewed in Jezek *et al*. 2018, 2023), their detection could provide valuable information for fertilization management and scientific analysis of plant responses to abiotic stress.

In our measurements, the Anth index values were quite low, never exceeding 0.15. The Anth index increased markedly under both N and Mg deficiency in leaf 3 (Fig. S2). However, anthocyanins could only be visually detected in Mg‐deprived plants (Fig. S1B), whereas N‐deficient leaves showed no leaf purpling (Fig. S1A). This indicates that the recorded Anth values are biased due to a methodological problem. This may be created by the used excitation wavelengths and declining Chl contents under stress conditions. In green control leaves, the Anth index increased with decreasing chlorophyll content (Fig. 3D,E). For the measurement of the Anth index, fluorescence is excited within the absorption maximum of anthocyanins and, for the reference, in the red region, where anthocyanins no longer absorb, but chlorophyll has a high absorption. Changing chlorophyll concentrations in a leaf will have a close to proportional effect on the absorption of green light by chlorophyll. However, in the red spectral region, at the high chlorophyll concentrations in a leaf, absorption is saturated and at first a change in concentration will have no or only a little effect on absorption. This effect was previously described as the package effect (Das *et al*. 1967; Osborne & Raven 1986). Nichelmann & Bilger (2017) showed that the fluorescence ratio upon excitation at 545 and 650 nm was dependent on chlorophyll concentration, using diluted chloroplast suspensions applied to a filter paper. At chlorophyll concentrations below 50 μg cm^−2^, which correspond to Dualex chlorophyll indices of 40 in maize leaves (Cerovic *et al*. 2012), the ratio *F*_545_/*F*_650_ started to decrease below the maximal value. This fits well with the chlorophyll index below which also in green leaves the anthocyanin index started to increase (Fig. 3D,E). However, in Mg‐deficient leaves, several data points did not follow the slow increase of the Anth values, but showed elevated values already at Chl indices of around 30. These data points were collected from seemingly purplish leaf parts (Fig. S3B), indicating that after proper correction for the effect of chlorophyll concentration on the Anth value, the latter could be used to quantify increased anthocyanin contents. Using anthocyanic hazelnut leaves, it has been shown that the Anth index detects independently determined anthocyanin contents with great precision (Bilger *et al*. 2026). For the practical use, a correction procedure requires that for the experimental plants, the relationship between the Anth and Chl indices is determined for visibly green leaves with varying chlorophyll content.

### Sugar induction of flavonoid synthesis

Accumulation of flavonoids in response to nutrient deficiency to sequester abundant carbon (C) is a prevailing hypothesis. For example, N deficiency‐induced anthocyanin accumulation in crops has commonly been explained by high foliar sugar levels (Bergmann 1993; Keller 2005). The relationship between N availability, plant growth and flavonoid biosynthesis is explained in the carbon‐nutrient balance hypothesis (Bryant *et al*. 1983; Nørbæk *et al*. 2003), which proposes that limited N availability restricts growth more than photosynthesis, resulting in an accumulation of C metabolites from carbon assimilation. Although speculative, it is proposed that excess C compounds are diverted into secondary metabolism to produce C‐based, N‐free compounds. This process is thought to reduce the risk of negative feedback inhibition of photosynthetic CO_2_ assimilation and over‐reduction of the photosynthetic electron‐transport chain in the thylakoid membranes, which can lead to excessive reactive oxygen species (ROS) formation. This assumption is supported by findings that external sugar feeding or disruption of phloem loading in source leaves induces genes of the flavonoid biosynthesis pathway (Lloyd & Zakhleniuk 2004; Teng *et al*. 2005; Solfanelli *et al*. 2006). However, the CNB hypothesis assumes a functionality that is speculative and has been criticized (Nitao *et al*. 2002). While high sugar levels can induce flavonoid accumulation, the exact function remains uncertain. Moreover, if one calculates the carbon sequestered in polyphenols under normal photosynthetic rates, the balance does not support this hypothesis considering the quantitative differences in anthocyanin production and sugar accumulation in leaves under nutrient deficiency. Anthocyanin concentrations only rise by a few hundred microgram per gram fresh weight (Juszczuk *et al*. 2004; Løvdal *et al*. 2010; Larbat *et al*. 2012), whereas sugar concentrations increase by several milligram per gram fresh weight under nutrient deficiency (Cakmak *et al*. 1994a; de Groot *et al*. 2003; Hermans *et al*. 2004). Thus, anthocyanins are probably no significant C sinks under nutrient deficiency. Similar conclusions were drawn in a study on the formation of chlorogenic acid derivatives in sunflower leaves under N deficiency (Negwer & Bilger 2026).

In our study, soluble sugars were markedly increased in leaves of Mg‐deficient maize seedlings especially in leaf 3 where concentrations rose up to 168 mg g^−1^ DW sucrose, 84 mg g^−1^ DW glucose and 45 mg g^−1^ DW fructose (Fig. 7). However, flavonols and anthocyanins in whole leaf extracts were in the range of few μg g^−1^ DW or even below the detection limit for HPLC analysis (data not shown). Considering these quantitative differences in sugar accumulation and flavonoid biosynthesis in nutrient‐deficient maize leaves, it can be assumed that flavonoids are not synthesized as C storage compounds in response to nutrient stress and might fulfil different ecophysiological functions such as attenuation of UV and photosynthetically active radiation and/or ROS scavenging (Gould 2004; Agati & Tattini 2010).

Accumulation of photoassimilates is a known phenomenon in response to Mg shortage and may arise from impaired phloem loading (Cakmak *et al*. 1994b; Verbruggen & Hermans 2013), or decreased metabolic activity in sink tissue (Fischer *et al*. 1998). Although soluble sugars accumulated in all analysed leaves at day 18, the Flav index was only significantly different from the control in leaf 3 and 4 under Mg deficiency (Figs 2 and 5C). Therefore, no correlative or causal relationship between the sugar accumulation and the Flav index can be deduced from the results. Low N supply increased glucose and sucrose concentrations in leaf 5 and leaves 3 and 4, respectively (Fig. 7A,C). Since the Flav index in all leaves under N deficiency was significantly higher than the control at harvest (Fig. 2), it suggests that sugar induction of flavonoid biosynthesis in response to N deficiency is possible. However, this interpretation requires careful differentiation. Strictly speaking, anthocyanins should also be considered, but the Flav index primarily measures flavonols and other simple polyphenols. Anthocyanins absorb less in the UV range than in the visible spectrum. Studies by Solfanelli *et al*. (2006) and Zirngibl *et al*. (2023) show that sugars (fructose, glucose, sucrose) primarily induce the anthocyanin branch, while early biosynthetic genes (EBGs) such as CHS and FLS1 are only moderately induced. The genetic and molecular regulation of nutrient deficiency‐induced flavonoid synthesis is poorly understood, especially in crops, and reports about sugar effects on gene expression are contradictory (Lillo *et al*. 2008). This differentiation is crucial for accurately interpreting the roles and regulation of these compounds in response to excess carbon. It has been suggested that sucrose is an essential factor for the induction of both flavonol and anthocyanin synthesis‐related genes (Stewart *et al*. 2001; Solfanelli *et al*. 2006; Lea *et al*. 2007; Olsen *et al*. 2008; Zhang *et al*. 2012; van den Ende & El‐Esawe 2014) involving sucrose non‐fermenting kinase 1 (SnRK1) during high‐light acclimation, a central regulator of energy and carbohydrate homeostasis in plants (Zirngibl *et al*. 2023). Also, glucose‐induction of major transcription factors for flavonoid biosynthesis has been reported (Zheng *et al*. 2024). Accumulated soluble sugars may thus serve as essential signalling elements in the genetic regulation of nutrient deficiency‐induced flavonoid synthesis, as it was reported for high‐light acclimation (Zirngibl *et al*. 2023).

## CONCLUSION

This study revealed that epidermal accumulation of UV‐A absorbing compounds is not an N deficiency‐specific stress response in maize seedlings but is also induced by Mg shortage, albeit to a clearly smaller extent and after extended time. Spatiotemporal monitoring of the Flav index along the leaf lamina revealed symptomatic differences between N and Mg deficiency. Whereas under N deficiency, UV‐A absorbing compounds accumulated especially in younger upper leaves, the Flav index increase started in Mg‐deprived plants at the tip of lower leaves. Both nutrient deficiencies decreased the NBI, but this was governed mainly by a loss of chlorophyll in Mg‐deprived plants, whereas under N deficiency, both an increase in the Flav index and a decrease in the Chl index were responsible for the changes in NBI. Therefore, not the NBI alone, but rather the relationship between both indices, Chl and Flav, can give an indication of the type of nutrient deficiency. In addition, early‐stage differentiation between N and Mg deficiency using the Dualex might be possible by measuring the Flav index in young, expanding tissue because differences between treatments were most apparent in these leaves. Measuring Chl fluorescence excited with green and red light provided indications of the formation of epidermal anthocyanin only when the influence of chlorophyll concentration on the anthocyanin index was taken into account.

## AUTHOR CONTRIBUTIONS

Conceptualization: MJ, C‐MG and K‐HM; Experimental work: MJ. Data analysis: MJ and WB; Visualization: MJ and MW. Original draft: MJ; Review and editing: MJ, K‐HM, C‐MG, ASR, MW and WB; All authors read and approved the final version of the manuscript.

## CONFLICT OF INTEREST

The authors declare that they have no conflict of interest.

## FUNDING INFORMATION

This work was funded by the Deutsche Forschungsgemeinschaft (DFG, German Research Foundation), project number 471624304. Mareike Jezek received a scholarship from the Heinrich‐Böll‐Foundation, Germany.

## Supporting information


**Fig. S1:** Leaves number 3 of 4 different 18 day old maize seedlings.
**Fig. S2:** Time course of Anth index of leaf 3.

## Data Availability

All the data supporting the article are available from the communicating authors upon due request.
