# Supplementary material for: Early‐stage diagnosis of nutrient deficiency by chlorophyll fluorescence in maize
Source: Plant Biol (Stuttg). 2026 Jul 31;28(6):1834–46. doi: 10.1111/plb.70273 (PMC13586641; doi:10.1111/plb.70273)
Supplement: Supplementary file 1 [file PLB-28-1834-s001.pdf]

Supplementary material

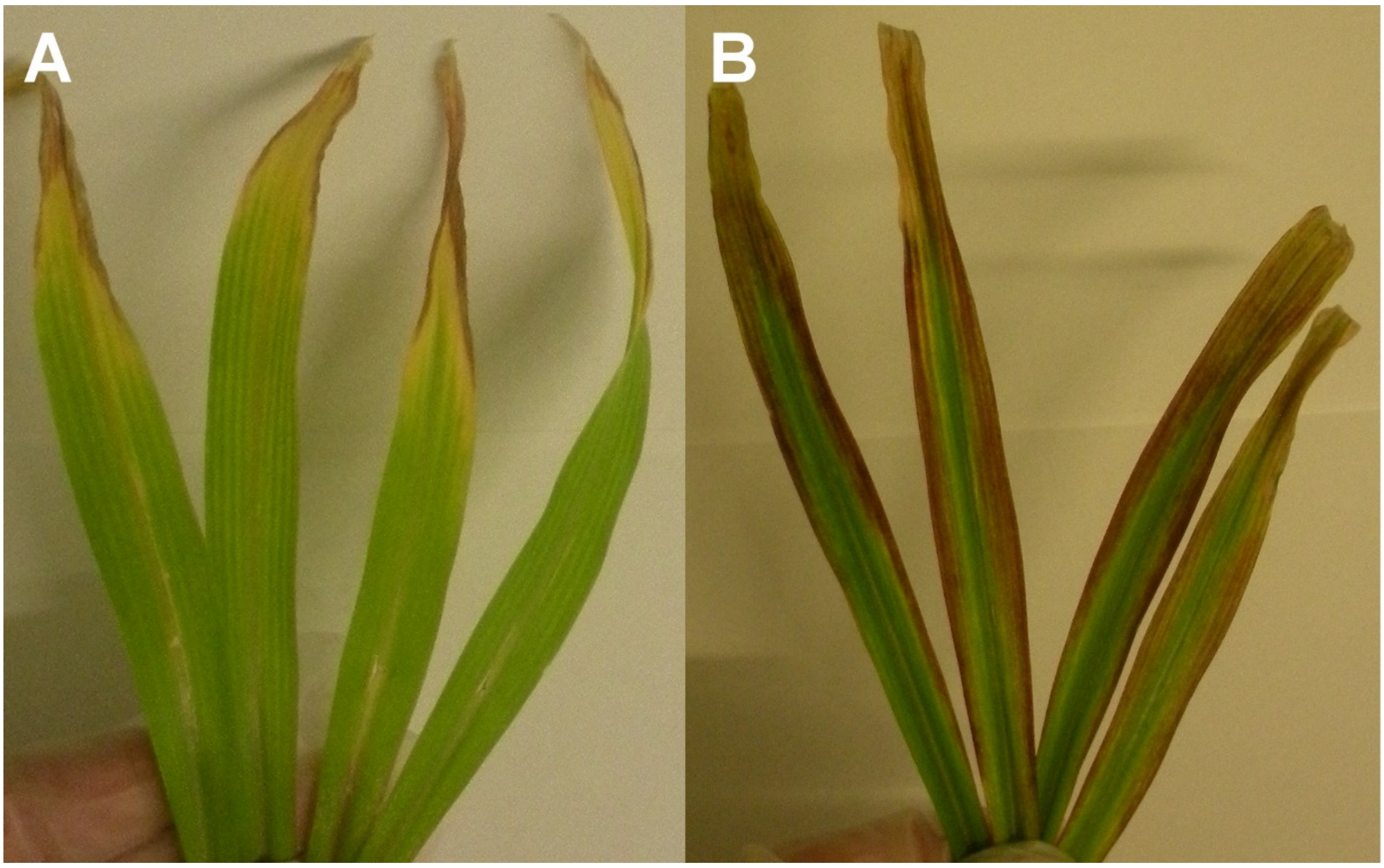

Figure S1: Leaves number 3 of 4 different 18-days-old maize seedlings grown under (A) N deficiency (4% N of the optimal N supply) and (B) Mg deficiency (4% of the optimal Mg supply). Nitrogen-deficient leaves showed chlorosis and leaf tip necrosis but no signs of purpling, whereas additional anthocyanin accumulation was visible under Mg shortage.

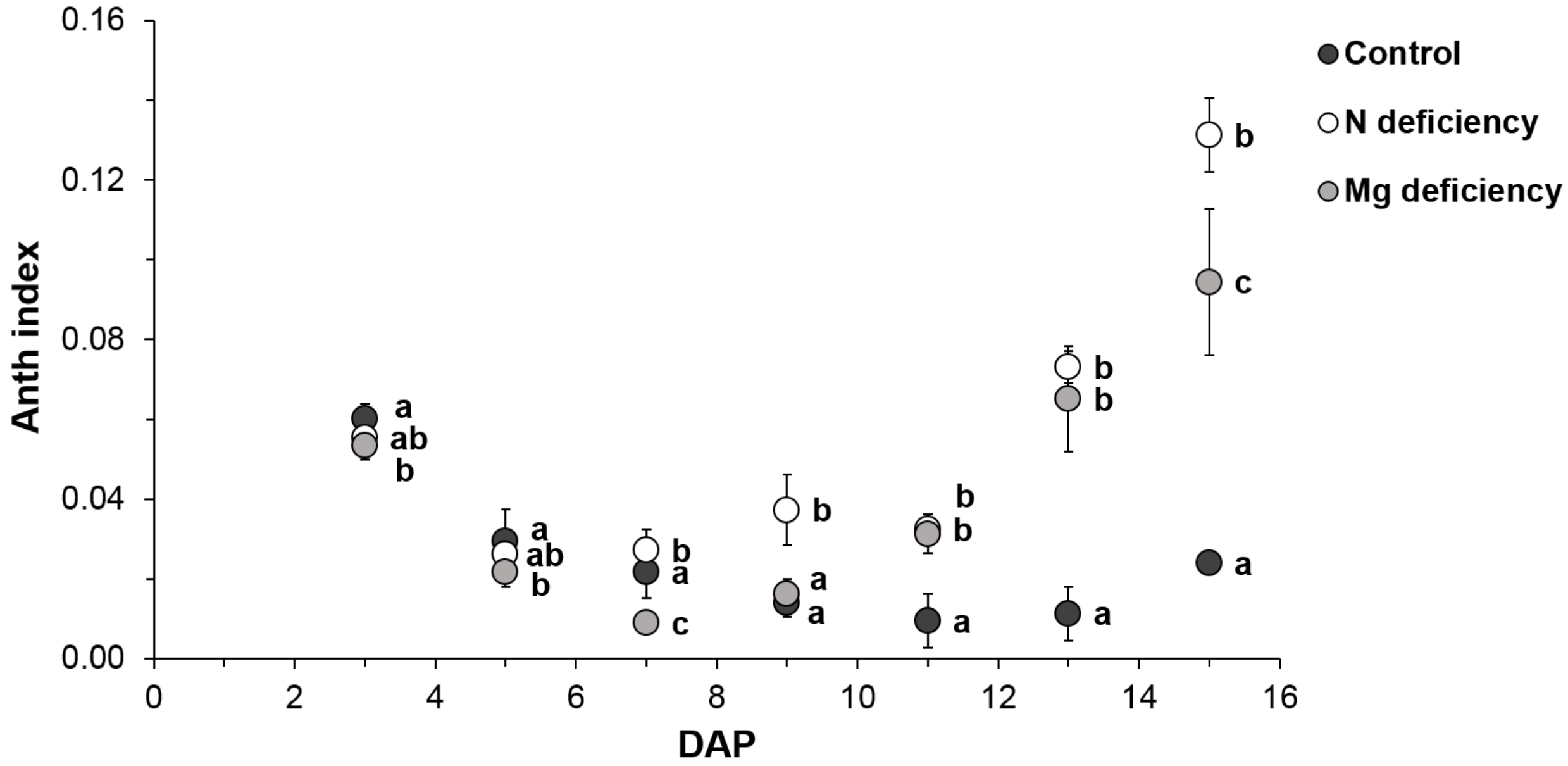

Figure S2: Time course of Anth index of leaf 3. Control plants were grown under optimal nutrition; N and Mg deficiency was induced by reducing the N or Mg concentration, respectively, in the nutrition solution to 4% of the control. Measurements were taken on four plants of four biological replicates per treatment. Five to six measurements were taken along the leaf blade and averaged. Mean ± SE; small letters indicate significant differences between treatments at a measuring date (pairwise t-test with Bonferroni adjustment, $p \leq 0.05$).
